# Transcriptome Analysis Reveals Unique Relationships Among *Eleusine* Species and Heritage of *Eleusine coracana*

**DOI:** 10.1534/g3.119.400214

**Published:** 2019-04-22

**Authors:** Hui Zhang, Nathan Hall, Leslie R. Goertzen, Charles Y. Chen, Eric Peatman, Jinesh Patel, J. Scott McElroy

**Affiliations:** * Department of Crop, Soil and Environmental Science; † Department of Biological Sciences, and; ‡ School of Fisheries, Aquaculture and Aquatic Sciences, Auburn University, Auburn, AL, 36849

**Keywords:** *Eleusine coracana*, *Eleusine africana*, transcriptome, relationships

## Abstract

Relationships in the genus *Eleusine* were obtained through transcriptome analysis. *Eleusine coracana* (*E. coracana* ssp. *coracana*), also known as finger millet, is an allotetraploid minor crop primarily grown in East Africa and India. Domesticated *E. coracana* evolved from wild *E. africana* (*E. coracana* ssp. *africana*) with the maternal genome donor largely supported to be *E. indica*; however, the paternal genome donor remains elusive. We developed transcriptomes for six *Eleusine* species from fully developed seedlings using Illumina technology and three *de novo* assemblers (Trinity, Velvet, and SOAPdenovo2) with the redundancy-reducing EvidentialGene pipeline. Mapping *E. coracana* reads to the chloroplast genes of all *Eleusine* species detected fewer variants between *E. coracana* and *E. indica* compared to all other species. Phylogenetic analysis further supports *E. indica* as the maternal parent of *E. coracana* and *E. africana*, in addition to a close relationship between *E. indica* and *E. tristachya*, and between *E. floccifolia* and *E. multiflora*, and *E. intermedia* as a separate group. A close relationship between *E. floccifolia* and *E. multiflora* was unexpected considering they are reported to have distinct nuclear genomes, BB and CC, respectively. Further, it was expected that *E. intermedia* and *E. floccifolia* would have a closer relationship considering they have similar nuclear genomes, AB and BB, respectively. A rethinking of the labeling of ancestral genomes of *E. floccifolia*, *E. multiflora*, and *E. intermedia* is maybe needed based on this data.

*Eleusine* is a small genus of annual and perennial grass species within the Eragrosteae tribe and Chloridoideae subfamily. It includes about 9 to 12 species that can hybridize to form intermediates and they are very similar in morphological features (Mehra 1962; Phillips 1972; Airy Shaw 1973; Hilu 1981). It is mainly distributed in the tropical and subtropical parts of Africa, Asia and South America (Phillips 1972). *Eleusine* contains diploid and tetraploid species, with chromosome numbers ranging from 2n = 16, 18 or 20 in diploids to 2n = 36 or 38 in tetraploids. All of the species are wild except *E. coracana*, which is cultivated for grain and fodder in Africa and the Indian subcontinent. The center of *Eleusine* diversity is East Africa and there are eight species in this genus occurring in this region, which includes *E. africana*, *E. coracana*, *E. kigeziensis*, *E. indica*, *E. floccifolia*, *E. intermedia*, *E. multiflora*, and *E. jaegeri* (Mehra 1963; Phillips 1972). The genome size of *Eleusine* species is very small and the 2C DNA amount ranges from 2.50 pg to 3.35 pg for diploid species (Hiremath and Salimath 1991). Questions remain regarding the evolutionary origins of the polyploid species and their relationship to wild diploid progenitors.

*E. coracana*, commonly referred to as finger millet or African finger millet, is the only domesticated *Eleusine*, which is cultivated as both grain and fodder primarily in semiarid regions of Africa and the Indian subcontinent (Bisht and Mukai 2001b). *E. coracana* is an allotetraploid species with a chromosome number of 2n = 4x = 36 that was reportedly domesticated from the wild tetraploid *E. africana* (2n = 4x =36) (Hilu and De Wet 1976; Dida *et al.* 2008). *E. coracana* is by all definitions an orphan crop, an important regional crop that lacks widespread use (Singh *et al.* 2014). Orphan crops also have societal benefits of aiding to sustain cultural richness and maintain community identity in rural societies (Naylor *et al.* 2004). Global climate change will have negative effects on the yield of major crops, which will conflict with increasing world population growth (Hisas 2011). In undeveloped regions of the world, continued failure to maintain increases in food production will lead to food price increases, as well as social unrest and famine (Abberton *et al.* 2016). Orphan crops such as finger millet could be a beneficial food source to ballooning world populations because they can be grown on more marginal land under harsher environmental conditions (Naylor *et al.* 2004).

The major limitation to developing orphan crops is that information on germplasm is not readily accessible and little information is found outside of traditional peer-reviewed academic publishing or written in languages not well-known to the scientific community concerned (Hammer and Heller 1998). In addition, existing knowledge on the genetic potential of minor crops is limited with few genetic resources, like genomes, transcriptomes and ESTs, available online compared to major or industrial crops (Dawson *et al.* 2009). Lack of information about origin and ancestry also inhibits breeding of minor crops. In plant breeding, paternal and maternal germplasm with desirable traits are collected and desirable traits are introduced to the cultivated species through hybridization and backcrossing (Simpson 2001; Chu *et al.* 2011). For example, knowing the parentage aided the development of peanuts since wild diploid *Arachis* species possess genetic variability in pest and disease resistance traits, which were used to improve cultivated peanuts (Stalker and Moss 1987; Chopra *et al.* 2016). Assessment of phylogenetic relationships is vital for any successful crop improvement since the wild relatives often have good traits and biodiversity.

With respect to the *Eleusine* genre, publicly available transcriptome assemblies have been produced for *E. indica* (Chen *et al.* 2015) and *E. coracana* (Rahman *et al.* 2014; Kumar *et al.* 2015), and 78 plastid protein coding loci were sequenced for *E. coracana* (Givnish *et al.* 2010). A complete chloroplast genome (Zhang *et al.* 2017) and a draft nuclear genome (Zhang *et al.* 2019) have been reported for *E. indica* and a draft nuclear genome has been reported for *E. coracana* (Hatakeyama *et al.* 2017; Hittalmani *et al.* 2017). Hatakeyama *et al.* (2017) used a novel multiple hybrid assembly workflow which is suitable for the assembly of complex allotetraploid species. Although there are more studies conducted for genomic resources of *E. coracana*, there is still only modest information on its evolution and progenitors. *E. indica*, an annual diploid (2n = 2x = 18), is most commonly mentioned as the maternal genome donor based on genomic *in situ* hybridization (Hilu 1988; Hiremath and Salimath 1992; Bisht and Mukai 2001a) although *E. tristachya*, a diploid (2n = 2x = 18) has not been eliminated as the maternal progenitor while *E. floccifolia*, a diploid (2n = 2x = 18) perennial species or an unknown or extinct ancestor is thought to be the paternal genome donor (Bisht and Mukai 2000, 2001a 2002; Liu *et al.* 2014). However, for these studies, the evidence was not enough since they only used one or few chloroplast genes or a single low copy nuclear gene as a marker. Thus, our objective was to provide a broader survey of *Eleusine* species evolutionary relationships based on separate analysis of chloroplast and nuclear transcriptomes and to verify the maternal genome donor of *E. coracana*.

## Materials and Methods

Germplasm was acquired from the U.S. National Plant Germplasm System (https://npgsweb.ars-grin.gov/gringlobal/search.aspx) Germplasm Resources Information Network (NPGS GRIN) for analysis. An exhaustive search for all available *Eleusine* species was conducted to identify all possible candidate species within the *Eleusine* genus. Seven of the nine known *Eleusine* species were identified and acquired for analysis (Table 1). *E. jaegeri* and *E. kigeziensis* were unavailable from NPGS GRIN. No other sources for these two species could be identified. A previously assembled transcriptome (Chen *et al.* 2015) and plastid genome (Zhang *et al.* 2017) of *E. indica* were utilized as references.

**Table 1:** Biological, genomic, and GRIN*^a^* Accession Number for seven *Eleusine* species utilized. Genomic and biological acquired from the following sources

Species	2n chromosome numbers, genome, ploidy	Life cycle	Type	GRIN Accession Number
*E. multiflora*	16, CC, diploid	Annual	Wild	226067
*E. floccifolia*	18, BB or other, diploid	Perennial	Wild	196853
*E. tristachya*	18 AA, diploid	Annual	Wild	331791
*E. intermedia*	18 AB, diploid	Perennial	Wild	273888
*E. africana*	36 AABB, allotetraploid	Annual	Wild	226270
*E. coracana*	36 AABB, allotetraploid	Annual	Cultivated	462949
*E. indica*	18 AA, diploid	Annual or Perennial	Wild	Collect*^b^*
*E. jaegeri*	20 DD, diploid	Perennial	Wild	Unavailable
*E. kigeziensis*	38 AADD, allotetraploid	Perennial	Wild	Unavailable

a GRIN, Germplasm Resources Information Network.

b *E. indica* was collected locally from a crop field in Tallassee, Alabama. In other published work by J.S. McElroy it is referred to by the acronym PBU referring to its origin at the Alabama Agricultural Experiment Station Plant Breeding Unit. *E. indica* is known to exist as a weedy perennial in managed ecosystems of southern Florida and Hawai’i.

*Eleusine* species were germinated and grown from seed in a glasshouse environment at 28 ± 2°, and 70% average relative humidity in Auburn, AL (32.35°N, 85.29°W). Seedlings were grown in a native Wickham sandy loam soil with pH 6.3 and 0.5% organic matter. Four-week old entire seedlings were used for RNA extraction. Total RNA was extracted from individual seedlings of *E. multiflora*, *E. floccifolia*, *E. tristachya*, *E. intermedia*, *E. africana*, and *E. coracana* using RNeasy Plant Mini Kit (Qiagen, CA, USA). The quality and quantity of total RNA were determined with gel electrophoresis and Nanodrop 2000 (Thermo Scientific). High-quality RNA was used for transcriptome sequencing.

RNA preparation and sequencing was conducted at the Genomic Service Laboratory at Hudson Alpha Institute for Biotechnology (Cummings Research Park, Huntsville, AL) using standard procedures for the Illumina HiSeq 2000 to produce 100 bp paired-end reads (Chen *et al.* 2015, 2016). One complementary DNA (cDNA) library was constructed for each of the six total RNA samples. All samples were subjected to polyA selection prior to sequencing. *E. indica* transcriptome (NCBI Accession No.: SRR1560465) previously assembled by our lab (Chen *et al.* 2015) was also sequenced by Hudson Alpha using the Illumina HiSeq 2000 platform and same methodology in the same growth conditions.

### Sequence data analysis and assembly

Raw reads quality were checked by FastQC v.0.11.1 software (Andrews 2010) and then processed by Trimmomatic v.0.33 (Bolger *et al.* 2014) to remove adapters and low quality reads and sequences. The trimmed reads were evaluated with FastQC again and normalized with Trinity’s in silico read normalization (Grabherr *et al.* 2011), with maximum coverage of 30. Three *de novo* transcriptome assemblers were used: Trinity v.2014-04-13p1 (Grabherr *et al.* 2011), Velvet v.1.2.08_ maxkmer101 (Zerbino and Birney 2008), and SOAPdenovo2 v.2.04 (Luo *et al.* 2012). Trinity k-mer size was 25. Velvet k-mer size was 21 to 91 with step size of 10 and minimum contig length was 200 bp without scaffolding. SOAPdenovo2 k-mer size was 21 and 31. The three *de novo* assemblers thus yielded 11 total assemblies for each species. The script Select_contigs.pl (https://pods.iplantcollaborative.org/wiki/display/DEapps/Select+contigs) was used for Trinity and SOAPdenovo2 to select contigs with minimum length 200 bp. To evaluate the quality of the assembly, N50s and contig length distributions of the assemblies were calculated with the script Count_fasta.pl (http://wiki.bioinformatics.ucdavis.edu/index.php/Count_fasta.pl). Before merging, “N”s were removed from the assemblies and contigs shorter than 200 bp were discarded.

**Figure 1:**
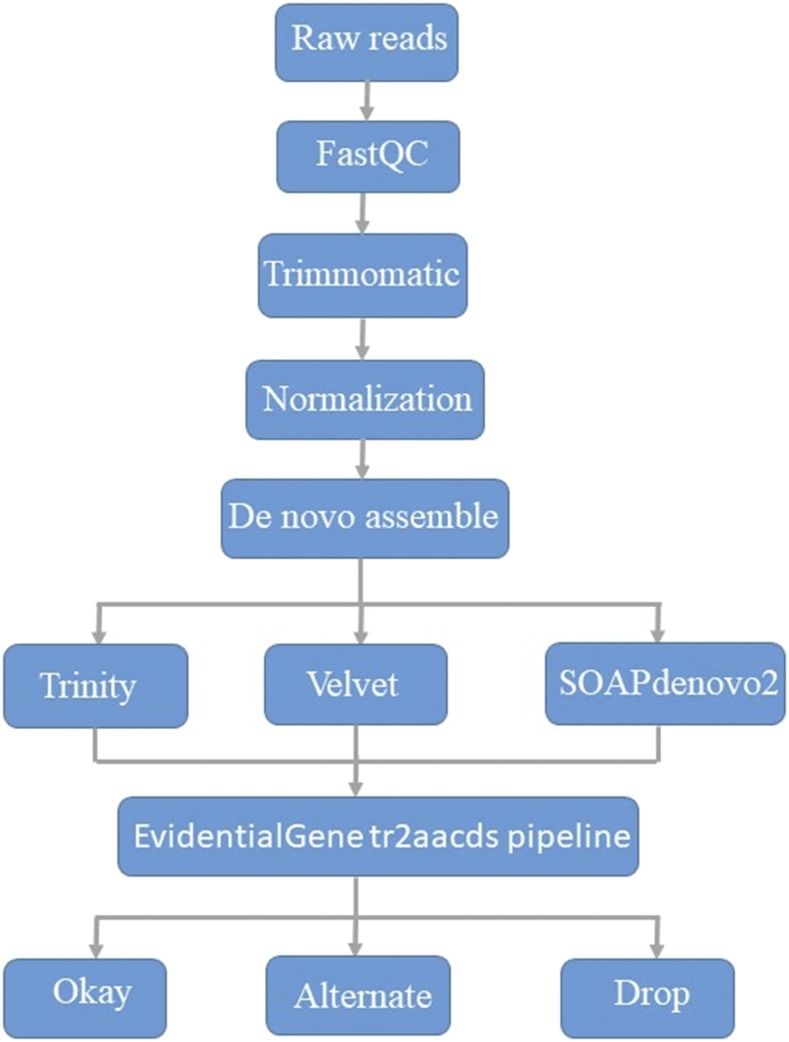
Workflow of transcriptome sequencing data analysis and assembly. Three *de novo* assemblers (Trinity, Velvet, and SOAPdenovo2) and a redundancy-reducing EvidentialGene tr2aacds pipeline were used for constructing optimized transcriptome references.

All assemblies were combined into one merged assembly for each species individually. The merged assembly was processed by EvidentialGene tr2aacds pipeline (http://arthropods.eugenes.org/EvidentialGene/about/EvidentialGene_trassembly_pipe.html). The EvidentialGene pipeline takes as input the transcript fasta file produced by any of the transcript assemblers and generates coding DNA sequences (CDSs) and amino acid sequences from each input contig then uses fastanrdb to quickly reduce perfect duplicate sequences, cd-hit and cd-hit-est to cluster protein and nucleotide sequences, and Blastn and makeblastdb to find regions of local similarity between sequences. It outputs transcripts into three classes: Okay (the best transcripts with the unique CDS, which is close to a biologically real set regardless of how many millions of input assemblies), Alternate (possible isoforms), and Drop (the transcripts did not pass the internal filter). The unique CDS (Okay set) and possible isoforms (Alternate set) were used for further evaluation and annotation. The overall workflow was summarized graphically in Figure 1.

### Annotation and analysis

Sequences were annotated using Trinotate v.2.02, which is a comprehensive annotation suite designed for automatic functional annotation of transcriptomes, particularly *de novo* assembled transcriptomes (Li *et al.* 2014). This pipeline includes: homology search to known sequence data (BLAST+/SwissProt), protein domain identification (HMMER/PFAM), protein signal peptide and transmembrane domain prediction (signalP/tmHMM), and leveraging various annotation databases (eggNOG/GO/Kegg databases). All functional annotation data derived from the analysis of transcripts are integrated into an SQLite database which allows fast efficient searching for terms with specific qualities related to a desired scientific hypothesis or a means to create a whole annotation report for a transcriptome. Blast2GO v.3.0 (Götz *et al.* 2008) was used to analyze the unique genes between *E. coracana* and *E. africana*.

### Variants analysis

Variants are mainly classified into five different types: single nucleotide variants (SNVs), multiple nucleotide variants (MNVs), insertions, deletions, and replacements. SNVs are one base replaced by another base, most commonly referred to as a single nucleotide polymorphism (SNP). MNVs are two or more SNVs in succession. Insertions are events where one or more bases are inserted in the experimental data compared to the reference. Deletions are events where one or more bases are deleted from the experimental data compared to the reference. Replacements are more complex events where one or more bases have been replaced by one or more bases, where the identified allele has a length different from the reference.

Read mapping and detection of SNVs, MNVs, replacements, insertions, and deletions were conducted using the tools ‘map reads to reference’ and ‘probabilistic variant detection’ separately in CLC Genomics Workbench v.6.5.2 (CLC Bio, Aarhus, Denmark). The mapping parameters were set to ‘Mismatch cost = 3, Insertion cost = 3, Deletion cost = 3, Length fraction = 0.95, Similarity fraction = 0.95’. The variants calling parameters were set to ‘Minimum coverage = 30, Variant probability = 90’.

### Chloroplast gene comparison

Complete *E. indica* chloroplast genome (KU833246) were downloaded from NCBI. The other *Eleusine* species’ CDS datasets were aligned to the chloroplast genome using Blastn at the E-value threshold 10^−5^, word size 20, and minimum match size 90. *E. coracana* reads were mapped to the aligned *Eleusine* species’ CDSs separately. SNVs, MNVs, replacements, insertions, and deletions were called from each of the mappings in CLC Genomics Workbench v.6.5.2 (CLC Bio, Aarhus, Denmark).

### Phylogenetic analysis

Two separate analyses were conducted to determine the potential parentage of *E. coracana*. First, chloroplast genome was compared among all *Eleusine* species, and second, transcriptomes of nuclear genes were compared among *Eleusine* species. Chloroplast genes of *E. indica* were downloaded from NCBI (KU833246), which was named *E. indica_cp* in phylogenetic tree. Chloroplast genes from *E. indica* transcriptome using blast method were obtained and named *E. indica_trans* in phylogenetic tree and we used this method to verify our result. TBLASTx was used to extract the best chloroplast genes from each *Eleusine* species separately. The results were checked with alignment viewer Seaview v.4 (Gouy *et al.* 2009) and adjusted to exclude any erroneous hits. A supermatrix of nucleotide sequence alignments was produced using FASconCAT-G_v1.02.pl (Kück and Meusemann 2010). Several steps were employed to extract the nuclear genes for phylogenetic analyses. The contigs were translated to coding protein sequences using Transdecoder v.3.0.1 (Ravin *et al.* 2016). The Python script reduce_protein_redundancy.py (https://github.com/mcelrjo/blastp_nr) was used to select the longest ORF to produce a set of unique sequences. Orthogroups were extracted and aligned from the set of unique sequences with Orthofinder v.1.1.8 (Emms and Kelly 2015). A concatenated supermatix was produced using FASconCAT-G_v.1.02.pl (Kück and Meusemann 2010). A codon by gene partition scheme was used in Partition-Finder v.2.0.0 (Lanfear *et al.* 2012) and model selection was limited to GTR-GAMMA and GTR-GAMMA+I with greedy search algorithm, and the best scheme was used for subsequent phylogenetic analysis. Individual nuclear gene alignments were reduced to include only representatives of Poaceae and cleaned with gBlocks v0.19b (Castresana 2000) using default settings. Both concatenated and individual nuclear gene trees were created using RAxML-MPI-AVX v.8.2.6 (Stamatakis 2014) with 100 rapid bootstraps, and GTRGAMMA model since RAxML employs only one model across all partitions per analysis. Trees were visualized with Figtree v.1.3.1 (Rambaut 2009).

### Comparative transcriptome analysis Between E. africana and E. coracana

Comparative transcriptome analyses were conducted with the following steps: 1) A list of unique protein-coding transcripts from the *E. coracana* transcriptome were compiled and queried against *E. africana* transcriptome; 2) For *E. coracana* contigs with no matches to the *E. africana* transcriptome assembly but with matches to the non-redundant database, the sequences of the top hits were retrieved from the non-redundant database and used to query the *E. africana* transcriptome assembly; 3) Those *E. coracana* transcripts that remained unidentified were identified as genes that were expressed in the *E. coracana*, but not expressed in the *E. africana*.

### Data availability

The sequencing reads of *E. multiflora*, *E. floccifolia*, *E. tristachya*, *E. intermedia*, *E. africana*, and *E. coracana* were deposited at NCBI Sequence Read Archive (SRA) database under the accessions SRR5467257, SRR5468569, SRR5468570, SRR5468571, SRR5468572, SRR5468573, respectively. Transcriptome Shotgun Assembly projects have been deposited at DDBJ/EMBL/GenBank under the accessions GGLR00000000, GGME00000000, GGMD00000000, GGMC00000000, GGMB00000000, and GGMA00000000, correspondingly. All of the versions described in this paper are the first version, GGLR01000000, GGME01000000, GGMD01000000, GGMC01000000, GGMB01000000, and GGMA01000000. Supplemental material available at FigShare: https://doi.org/10.25387/g3.7994039.

## Results and Discussion

### Transcriptome sequencing and de novo assemblies

Read counts before and after quality checking and trimming are presented in Table 2. The summary statistics of the assemblies from EvidentialGene tr2aacds pipeline are shown in Table 3. Previous research has demonstrated this pipeline to improve transcript integrity and reduce assembly redundancy in transcriptome assembly (Chen *et al.* 2015). Average read length after trimming was 99.3 to 99.4 nucleotides. The N50 of the unique CDS set ranged from 1,471 to 1,693; however, when the possible isoform set is added, the N50 ranged from 1,232 to 1,451.

**Table 2:** The number and average length of *Eleusine* transcriptome sequencing reads before and after trimming

Species	Number of reads	Average length	Number of reads after trim	% reads removed	Average length after trim
*E. multiflora*	61,348,758	100	52,236,532	15%	99.4
*E. floccifolia*	59,140,884	100	50,053,954	15%	99.4
*E. tristachya*	53,661,434	100	45,004,810	16%	99.4
*E. intermedia*	106,867,304	100	84,798,308	21%	99.4
*E. africana*	197,003,984	100	156,392,016	21%	99.3
*E. coracana*	139,928,698	100	111,917,028	20%	99.3
*E. indica*	230,466,942	100	183,323,866	17%	99.4

**Table 3:** Summary statistics of transcriptome assemblies following implementation of N50, sequences number, and total length in EvidentialGene tr2aacids pipeline

Species	Unique CDSs			Unique CDSs + Possible isoforms		
	N50 (bp)	Sequences number	Total length (bp)	N50 (bp)	Sequences number	Total length (bp)
*E. multiflora*	1567	30,394	32,083,609	1357	52,610	50,466,628
*E. floccifolia*	1585	36,364	37,932,847	1361	72,602	69,442,718
*E. tristachya*	1549	35,856	37,243,265	1353	72,764	69,722,866
*E. intermedia*	1693	39,540	43,739,409	1451	87,270	87,954,199
*E. africana*	1516	56,375	54,910,276	1236	144,921	129,354,728
*E. coracana*	1471	59,223	561,062,47	1232	144,460	128,133,958
*E. indica*	1562	25,878	28,239,951	1408	36,959	37,055,659

For annotation, unique CDS assemblies of each transcriptome set were initially assigned with Trinotate v.2.02. GoTermParse.py (https://gist.github.com/NDHall/) was used to retrieve GO Terms and three components (Table S1). GoTermParse.py used regular expressions and a dictionary to sort terms into their major functional groups. The GO classification assigned totals of 516,793; 634,349; 578,631; 803,545; 996,369; 1,039,581; and 276,976 GO terms to *E. multiflora*, *E. floccifolia*, *E. tristachya*, *E. intermedia*, *E. africana*, *E. coracana*, and *E. indica* unique CDS set, respectively. All of the GO terms in *E. coracana* ‘unique CDS’ set have higher scores than in others. Integral_component_of_membrane, transcription_DNA-templated and ATP_binding are the highest GO terms in each corresponding component (Figure S1).

### E. coracana maternal genome donor

In order to elucidate the maternal genome donor of *E. coracana*, *E. coracana* reads were mapped to the assembled and identified chloroplast genes of *E. multiflora*, *E. floccifolia*, *E. tristachya*, *E. intermedia*, *E. africana*, *E. coracana*, and *E. indica*, respectively. *E. coracana* reads were also mapped to its own assembled and identified chloroplast genes (Table 4). Since some chloroplast genes have no hit for some species when they do Blast, the genes shared by all of the species were used. The name and type of chloroplast genes are summarized in Table 5. A total of 238,136; 246,733; 234,583; 226,923; 248,315; 225,962; and 249,884 reads were mapped to chloroplast genes of *E. multiflora*, *E. floccifolia*, *E. tristachya*, *E. intermedia*, *E. africana*, *E. coracana*, *and E. indica*, respectively, and covered 37,056; 38,012; 34,937; 36,287; 40,171; 37,969; and 42,162 bp of the references, respectively (Table 4). The variants (SNVs, MNVs, replacements, insertions, and deletions) detected from the *E. coracana* reads mapping to the chloroplast genes of *Eleusine* species were calculated. The least total variants across all variant types were mapping of *E. coracana* reads to *E. coracana* chloroplast genes. Excluding *E. coracana* and *E. africana*, *E. indica* had lower variants when *E. coracana* reads mapped to chloroplast genes of all *Eleusine* species, followed by *E. tristachya*. The detection of variants between reads of *E. coracana* and other *Eleusine* species in maternally inherited chloroplast further substantiated *E. indica* as the maternal genome donor. Further, this analysis gave us our first indication of a unique possible relationship between *E. coracana*, *E. africana*, *E. indica*, and *E. tristachya* simply based on the lower number of variants that occurred compared to other species.

**Table 4:** The mapped reads, covered references, mapped percentage and the length of SNVs, MNVs, replacements, insertions, and deletions detected from the *E. coracana* reads mapped to the chloroplast genes of all *Eleusine* species

Assembled species	Mapped reads	Covered reference*^a^*	Mapped percentage	SNVs	MNVs	Replacements	Insertions	Deletions
*E. coracana*	225,962	37,969	0.2%	15	0	0	0	0
*E. multiflora*	238,136	37,056	0.2%	106	0	0	0	0
*E. floccifolia*	246,733	38,012	0.2%	80	0	0	0	0
*E. tristachya*	234,583	34,937	0.2%	41	0	0	2	0
*E. intermedia*	226,923	36,287	0.2%	364	0	1	1	1
*E. africana*	248,315	40,171	0.2%	14	1	0	2	2
*E. indica*	249,884	42,162	0.2%	33	0	0	0	3

a The length of covered reference is similar but not same, because some chloroplast gene sequences are not exactly same.

**Table 5:** The summary of chloroplast genes used for determination of maternal genome donor of *E. coracana*

Category	Group	Gene name
Photosynthesis	Subunits of NADH-dehydrogenase	*ndhA*, *ndhB*, *ndhD*, *ndhE*, *ndhF*, *ndhG*, *ndhH*
	Subunits of photosystem I	*psaA*, *psaB*
	Subunits of photosystem II	*psbA*, *psbB*, *psbC*, *psbD*
	Subunits of cytochrome b/f complex	*petA*
	Subunits of ATP synthase	*atpA*, *atpB*, *atpE*, *atpI*
	Large subunit of rubisco	*rbcL*
Replication	Small subunit of ribosome	*rps2*, *rps4*, *rps7*, *rps11*, *rps12*, *rps19*
	Large subunit of ribosome	*rpl2*
	DNA dependent RNA polymerase	*rpoA*, *rpoB*, *rpoC1*, *rpoC2*
Other	Maturase	*matK*
	Protease	*clpP*
	c-type cytochrome synthesis gene	*ccsA*

**Figure 2:**
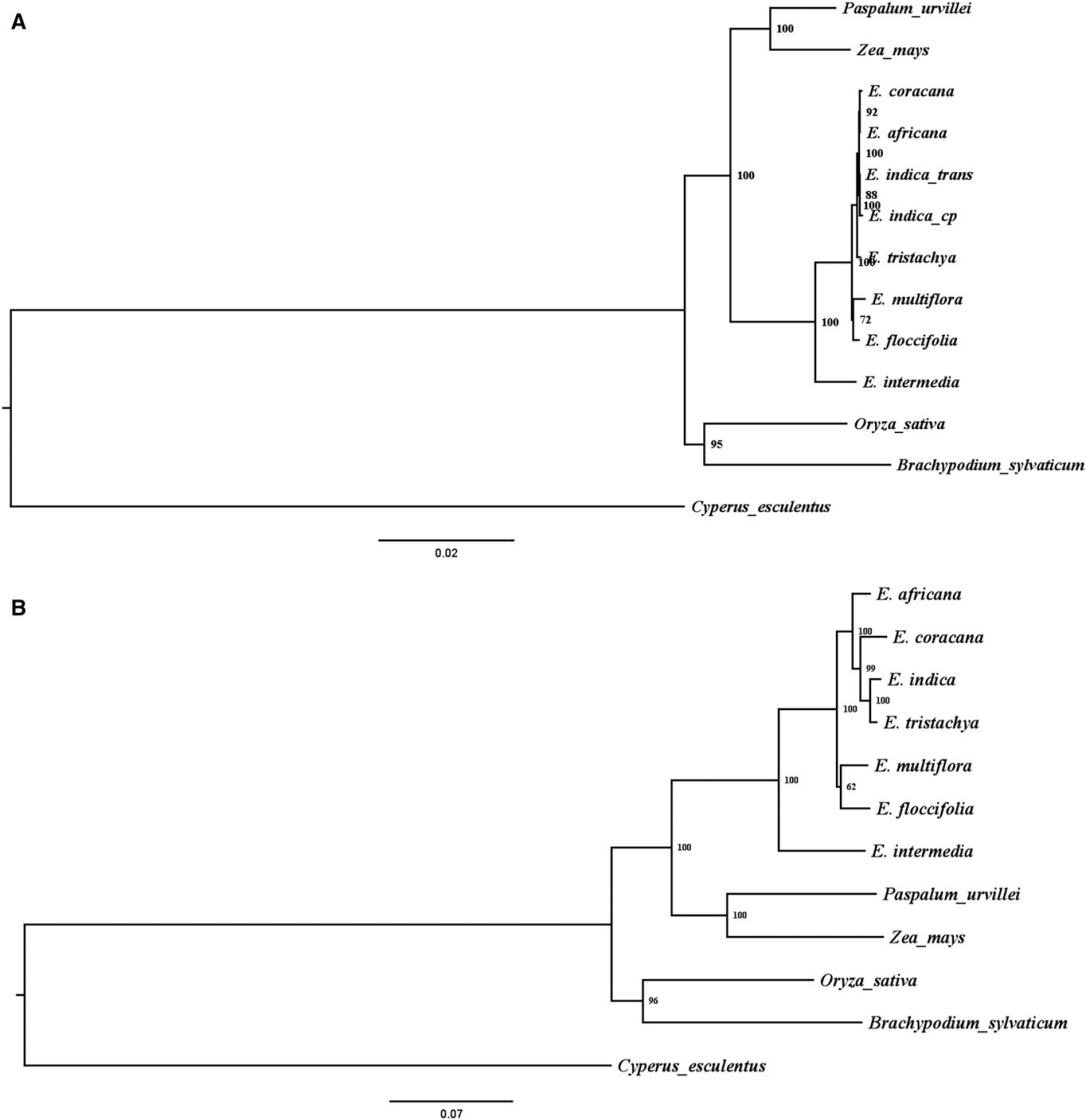
(A) Phylogenetic tree made using concatenated chloroplast genes in RAxML. Chloroplast genes of *E. Indica*_cp means these genes downloaded from NCBI (KU833246), which were accurate assembled and uploaded before. However, genes of *E. indica*_trans were got using same blast method with other species and we can also use this method to verify our result. (B) Phylogenetic tree constructed based on orthologous genes.

Concatenated phylogenetic trees were rooted using chloroplast and ortholog genes separately (Figure 2A, 2B). In the chloroplast gene derived tree, *E. coracana*, *E. africana*, and *E. indica* formed a clade that is sister to *E. tristachya*. A close phylogenetic relationship of *E. coracana*, *E. africana*, and *E. indica* further supports the hypothesis of *E. indica* as the maternal genome donor to the crop species *E. coracana*. Nuclear gene tree analyses eliminate *E. floccifolia*, *E. intermedia*, and *E. multiflora* as potential maternal genome donors with high bootstrap support. It does not eliminate *E. indica* or *E. tristachya* as a potential maternal genome donor. Our use of single copy genes from an allotetraploid that may have differences in homeologous gene expression limits the conclusions that can be drawn. To better understand the contributions of each subgenome to the super-matrix, subgenome identity was also predicted from individual gene tree topology (Figure S2). These results support *E. indica* as the maternal genome donor of *E. coracana* and again a close relationship between *E. indica* and *E. tristachya*, and also between *E. floccifolia* and *E. multiflora*. Our maternal genome donor conclusions are consistent with approaches such as genomic *in situ* hybridization (GISH), cytogenetic analysis, and phylogenetic analysis that conclude *E. indica* is the maternal parent of *E. coracana* (Bisht and Mukai 2001a, 2001b). Hatakeyama *et al.* (2017) also constructed a molecular phylogenetic analysis using two low-copy-number genes in *E. coracana* and concluded that *E. indica* was close to *E. coracana*, consistent with our phylogenetic analysis. Chloroplast DNA is highly conserved and its potential usefulness in phylogenetic studies has been well documented (Curtis and Clegg 1984; Palmer 1985; Hilu 1988). Here, we broadened the *E. coracana* maternity analysis to all assembled chloroplast genes in all our *Eleusine* transcriptome profiles. In addition, a close relationship between *E. floccifolia* and *E. multiflora* was supported by both of the phylogenetic trees. This relationship has been reported by Neves *et al.* (2005) using *trnT-trnF* region of plastid DNA, by Liu *et al.* (2011) using nuclear *EF-1a* data and by Hatakeyama *et al.* (2017) using phosphoenolpyruvate carboxylase 4 (*Pepc4*) gene.

### Comparative subtraction of the E. africana transcriptome from the E. coracana transcriptome

*E. africana* is considered to be the wild progenitor of domesticated *E. coracana* (Bisht and Mukai 2002). To provide insights into the genomic causes for the evolution in *E. coracana*, comparative transcriptome analysis (single replication of each species only) between *E. africana* and *E. coracana* was conducted, allowing identification of 2,737 genes that were expressed only in *E. coracana* but not in *E. africana*. Phylogenetic analysis (Figure 2A) also indicated *E. indica* was the maternal genome donor for *E. africana*. These data indicate that *E. indica* and *E. tristachya* possess a close relationship to *E. africana* and *E. coracana*. As such, *E. africana* might be autotetraploid species from *E. indica* genome doubling or through hybridization between *E. indica* and *E. tristachya*. However, such a conclusion is only based on this research, as more evidence using genomic sequencing would be needed to support such a hypothesis. Moffett and Hurcombe (1949) first reported *E. africana* from Africa as a tetraploid form of *E. indica*. Phylogenetic analyses of *E. coracana* genome (Hatakeyama *et al.* 2017) also indicated that *E. indica* and *E. tristachya* were in the same clade with *E. africana* and *E. coracana*, which is consistent with the results in this research.

### Conclusions

In this study, we constructed optimized transcriptome references for *E. multiflora*, *E. floccifolia*, *E. tristachya*, *E. intermedia*, *E. africana*, and *E. coracana* and the relationships among *Eleusine* species were investigated. By comparing the chloroplast genes among *Eleusine* species, we demonstrated that *E. indica* is the maternal genome donor and a maternal relationship exists between *E. indica* and *E. tristachya*. It is traditionally accepted that *E. coracana* evolved from the *E. africana* (Hilu and De Wet 1976) and is substantiated by more recent research (Dida *et al.* 2008). Transcriptomes are made publicly available for comparison to other species and to aid in identifying the paternal genome donor. Abundant *Eleusine* genetic resources from this research will be useful for the continued study of *Eleusine* evolution.
